# Differentiating Occupational Burnout Among Chinese Nurses: Moderating Roles in Nursing Work Environment and Perceived Care Quality

**DOI:** 10.3390/healthcare12222201

**Published:** 2024-11-05

**Authors:** Yucheng Cao, Qiwei Wu, Leiyu Shi, Yu Gao, Kathy Chappell, Jing Shao

**Affiliations:** 1 Johns Hopkins Bloomberg School of Public Health, Baltimore, MD 21205, USA; ycao31@jh.edu (Y.C.); lshi2@jhu.edu (L.S.); 2 Institute of Nursing Research, Zhejiang University School of Medicine, Hangzhou 310058, China; 12318339@zju.edu.cn; 3 School of Informatics, Computing, and Cyber Systems, Northern Arizona University, Flagstaff, AZ 86011, USA; yg542@nau.edu; 4 Accreditation Commission for Education in Nursing, Atlanta, GA 30326, USA; kchappell@acenursing.org

**Keywords:** nurses, occupational burnout, nursing work environment, perceived care quality, latent profile analysis

## Abstract

**Objective**: To investigate and identify different profiles of occupational burnout among Chinese nurses and explore how these burnout profiles moderate the relationship between the nursing work environment and perceived care quality. **Methods**: Cross-sectional data retrieved from the Chinese Nursing Work Environment Survey (C-NWES) were used for analysis. Variables of interest included occupational burnout, the nursing work environment, nurses’ perceived care quality, and demographic characteristics. Latent profile analysis was employed to identify potential profiles of occupational burnout, which were subsequently used as moderating variables to explore the relationship between the nursing work environment and perceived care quality. **Results**: Four profiles of occupational burnout were identified among Chinese nurses: Self-Fulfillment profile (38.3%), Emotional Exhaustion profile (37.7%), Lack of Accomplishment profile (16.6%), and Self-Isolation profile (7.4%). Among these, nurses in the Self-Fulfillment profile showed the greatest responsiveness to changes in the work environment, followed by those in the Self-Isolation profile, with the Emotional Exhaustion profile being the least responsive. **Conclusions**: The findings highlight the need for tailored interventions to address the specific needs of distinct nurse groups experiencing burnout, enabling organizations to improve nurses’ well-being, work performance, and patient care quality.

## 1. Introduction

Occupational burnout is a significant concern within the nursing profession; it is characterized by a state of physical, emotional, and mental exhaustion resulting from chronic exposure to job stressors [1]. Globally, the prevalence of nurse burnout is estimated to be as high as 30%, with significant regional differences [2]. Compared to international data, burnout among Chinese nurses is particularly pronounced. For example, studies indicate that about 68.1% of Chinese nurses have experienced burnout, and the prevalence is similarly high, at 64%, in hospitals in East China, far exceeding the global average [3,4]. This high prevalence not only emphasizes the severity of occupational burnout among Chinese nurses but also reveals its potential negative impact on the future development of the nursing workforce and the quality of patient care.

In response to the pressing issue of occupational burnout, numerous scholars have investigated both the phenomenon itself and its influencing factors, with the aim of developing effective, targeted strategies to alleviate burnout. Typically, occupational burnout can be divided into three dimensions: emotional exhaustion, depersonalization, and reduced personal accomplishment. These form the core content of the Maslach Burnout Inventory (MBI) [5]. A systematic review indicates that, over the past decade, the prevalence of high emotional exhaustion, high depersonalization, and low personal accomplishment in nursing has reached 31%, 36%, and 29%, respectively [6]. Although these studies report the prevalence of burnout dimensions, they often neglect the subtle differences among various burnout profiles, which limits their ability to guide personalized intervention strategies [7]. According to the multidimensional theory of burnout, different characteristics of burnout may reflect complex interactions among various influencing factors, necessitating further exploration through more refined classification methods [8].

As research advances, person-centered techniques like latent profile analysis (LPA) provide methods to identify individual differences in occupational burnout [9]. For instance, Rink et al. used LPA to identify several burnout profiles among nurses, such as “exhausted”, “exhausted with thriving”, “exhausted with thriving and recovery”, and “thriving and recovery” [10]. This classification suggests that nurses experiencing high burnout levels may struggle to provide high-quality care due to emotional exhaustion and depression. Additionally, a cluster analysis identified five profiles along the burnout–engagement continuum [11]. Although these studies provide insights into different characteristics of nurse burnout, they are constrained by small sample sizes and inconsistent results, highlighting the need for further exploration through large-scale surveys. Meanwhile, numerous studies over the past two decades have shown that a healthy nursing work environment—characterized by strong teamwork, organizational support, and adequate staffing—can effectively reduce burnout levels and improve care quality [12]. Conversely, a lack of supportive work environments can exacerbate stress, resulting in higher burnout levels and negatively impacting care quality [13]. For example, Omidi et al. demonstrated that high workloads in neonatal intensive care units (NICUs) not only increased burnout among nurses but also adversely affected their quality of life and job performance [14]. Furthermore, extensive research has shown that burnout affects not only nurses’ well-being but also patient care quality, organizational efficiency, and healthcare costs [15,16]. However, most existing studies tend to treat burnout as a singular variable, focusing on its interacting role in the relationship between the nursing work environment and patient care quality, while lacking exploration of how different nurse groups with varying burnout characteristics may respond differently to these relationships. Therefore, future research should focus on the relationship between different burnout profiles and nurses’ perceptions of their work environment and care quality to better understand these interactions and develop effective interventions.

Thus, the aim of this study was to analyze the profiles of occupational burnout among nurses in mainland China and explore how these burnout profiles moderate the relationship between the nursing work environment and nurses’ perceived quality of care. Therefore, this study aimed to explore potential profiles of occupational burnout among nurses in mainland China and explore the interactive effects of these profiles on the relationship between the nursing work environment and nurse-perceived care quality. We hypothesized that (1) occupational burnout among nurses can be categorized into distinct profiles, each with significant differences in demographic variables and that (2) different burnout profiles exhibit varying moderating effects on the relationship between the nursing work environment and perceived care quality. The anticipated findings will not only provide deeper insights into the categories and prevalence of occupational burnout among Chinese nurses but also offer international reference for developing targeted and personalized management strategies to alleviate burnout and improve care quality, thereby providing valuable guidance for global nursing practice.

## 2. Materials and Methods

### 2.1. Study Design and Subjects

This study is a secondary analysis based on the Chinese Nursing Work Environment Survey (C-NEWS), which was conducted in 2017 [17]. Nurses across the country were invited to participate by completing the questionnaires via a medical social media platform. Participants accessed the online questionnaire through a QR code or link. All procedures adhered to the ethical standards of the Declaration of Helsinki and the National Research Committee and were approved by the Ethics Committee of Sir Run Run Shaw Hospital, affiliated with Zhejiang University. Finally, the survey included registered nurses from medical institutions across 31 provinces, covering eight major economic regions in China, ensuring broad national representation. A total of 19,184 nurses participated, reflecting diverse healthcare settings throughout the country. Participants included in this study met the following criteria: (1) being a registered nurse; (2) having more than three months of work experience; and (3) currently working in frontline clinical settings. The variables of interest included nurses’ demographic information, nursing work environment, burnout, and perceived quality of care. The secondary analysis for this study received approval from the Ethics Committee of the Johns Hopkins Bloomberg School of Public Health, ensuring compliance with ethical guidelines for data use and research conduct.

### 2.2. Measures

#### 2.2.1. Nurse Occupational Burnout

The Chinese version of the Maslach Burnout Inventory (MBI) was used to measure nurses’ occupational burnout. This scale was originally developed by Maslach and Jackson (1981) and later culturally adapted for the Chinese context by Luo Hong et al. [8,18]. The Chinese version of the MBI scale comprises 22 items across three dimensions: emotional exhaustion, depersonalization, and personal accomplishment. A seven-point Likert scale was used to indicate the frequency of various feelings experienced by nurses, with responses ranging from “never” (zero points) to “every day” (six points). The Cronbach’s alpha coefficients for emotional exhaustion, depersonalization, and personal accomplishment in this study were 0.875, 0.761, and 0.848, respectively [18].

#### 2.2.2. Nursing Work Environment

The nursing work environment was measured using the Chinese Nursing Work Environment Scale (C-NEW scale) developed by the research team based on a framework that integrates key elements of the nursing work environment and the theory of harmony between individuals and their environment [17]. The scale is specifically designed for the Chinese cultural context and consists of 26 items across seven dimensions: professional development, leadership and management, nurse–physician relationship, recognition of value, clinical autonomy, basic benefits, and staffing adequacy. A six-point Likert scale was used to represent “very dissatisfied” (one point) to “very satisfied” (six points). Previous studies have demonstrated the scale’s reliability and validity. The Cronbach’s coefficient for the scale was 0.97, with coefficients for the subscales ranging from 0.77 to 0.97 in this study [19].

#### 2.2.3. Nurses’ Perceived Care Quality

Nurses’ perceived care quality was measured using a two-item scale extracted from the Nursing Quality Report, which has demonstrated high reliability [20,21]. The items assessed the quality of nursing care at the hospital where the nurses were employed and the quality of care they provided during their most recent shift. The responses were ranked using a four-point scale, from one (excellent) to four (poor) [20].

#### 2.2.4. Demographic Characteristics

A general demographic information questionnaire was utilized to collect variables such as gender, age, years of service, marital status, educational level, position, professional title, and economic region of origin.

### 2.3. Statistical Analysis

Latent profile analysis was conducted using Mplus 8.0 to identify potential categories of occupational burnout among nurses in mainland China. The analysis proceeded from one category to five categories, with each model being evaluated based on its fit to the data and empirical evaluations. The evaluation metrics included the Akaike information criterion (AIC), the Bayesian information criterion (BIC), the entropy value, the Lo–Mendell–Rubin test (LMRT), and the bootstrapped likelihood ratio test (BLRT) [22,23]. Lower AIC and BIC values indicate a superior model. The entropy value is used to assess the quality of model classification, with values closer to 1 indicating a superior model. The LMRT is employed to ascertain the optimal number of categories, assuming that a model with k categories is superior to a model with k−1 categories when *p* < 0.05. The BLRT is utilized to assess whether models with differing numbers of categories demonstrate a significant improvement in fit, with *p* < 0.05 serving as the threshold for significance. The ggplot2 package in R 4.2.2 was used to generate the graphical representation of the latent profile analysis results.

Subsequent analysis after LPA was conducted using SPSS 26.0. Continuous variables were represented by means ± standard deviations or medians and quartiles, while categorical data were described by frequencies and proportions. Chi-square tests were conducted on different demographic data across subgroups, and variables with statistical significance (*p* < 0.05) were included as control variables in subsequent moderation analyses. The Kruskal–Wallis H test was employed for group comparisons, with pairwise comparisons conducted using the Bonferroni method [24]. The data on the work environment and nurse-perceived care quality were standardized, and a moderation analysis was conducted using the PROCESS macro (model 1).

## 3. Results

### 3.1. Participants’ Demographic Characteristics

Overall, 16,382 participants were included in the analysis. Among them, there were 14,754 women (90.1%) and 1628 men (9.9%), with an average age of 30.39 ± 6.76 years and an average of 8.43 ± 7.30 years of work experience. A total of 10,818 participants (66.1%) had a bachelor’s degree or higher, and 6344 (38.7%) held intermediate professional titles.

### 3.2. Latent Profile Analysis Results of Occupational Burnout

The findings from the LPA of occupational burnout among Chinese nurses indicated that as the number of categories increased, the AIC and BIC values gradually decreased. However, the highest entropy value was observed in the four-class categorization, indicating that boundary delineation was more identifiable. Based on clinical management experience, as well as an assessment of the data distribution and differentiation between different profiles, the four-class categorization was determined to be the optimal model, as shown in Table 1.

**Figure 1:**
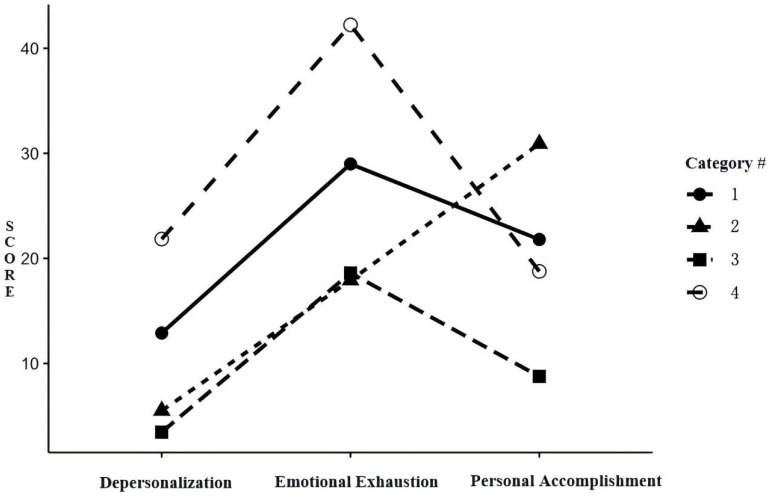
Latent profile analysis categories of nurse occupational burnout (note: in the dimensions of emotional exhaustion and depersonalization, higher scores indicate more severe fatigue. In contrast, the Personal Accomplishment dimension uses reverse scoring, so higher scores represent lower levels of burnout).

The differences in scores across the three dimensions of occupational burnout among the different categories were compared, as shown in Table 2. The scores were then plotted to classify the latent profiles (Figure 1). Category 1 (accounting for 37.7% of the total participants), characterized by high depersonalization, low personal accomplishment, and slightly elevated work fatigue, was identified as the “Emotional Exhaustion” profile. Category 2 (accounting for 16.6% of the total participants), which showed occupational burnout primarily in the dimension of personal accomplishment, with the highest scores in this dimension, was identified as the “Lack of Accomplishment” profile. Category 3 (accounting for 38.3% of the total participants) demonstrated a low fatigue level across all aspects coupled with the lowest depersonalization and emotional exhaustion scores. This suggested a willingness to engage with service recipients and attain a sense of accomplishment in nursing work; thus, it was named the “Self-Fulfillment” profile. Lastly, Category 4 (accounting for 7.4% of the total participants), characterized by high occupational burnout across all dimensions and particularly high burnout in depersonalization and emotional exhaustion, was identified as the “Self-Isolation” profile.

### 3.3. Demographic Characteristics of Different Occupational Burnout Categories

The demographic profiles of nurses across different occupational burnout categories are shown in Table 3. The findings revealed significant differences in age, years of service, gender, marital status, educational level, shift method, position, professional title, and economic region of origin among nurses across different occupational burnout profiles (*p* < 0.05). Specifically, Self-Fulfillment nurses are mainly clinical nurses over 35 years old with more than five years of work experience and holding a bachelor’s degree or lower. Over half of male nurses belong to the Emotional Exhaustion profile. Nurses with Lack of Accomplishment are mostly clinical nurses with less than five years of work experience; they exhibit significant burnout in the dimension of personal accomplishment. Self-Isolation nurses mainly work night shifts, most are between 25 and 35 years old, and many come from the eastern coastal regions.

### 3.4. Effects of Occupational Burnout Categories on the Relationship Between Work Environment and Perceived Care Quality at Hospital and Unit Level

**Figure 2:**
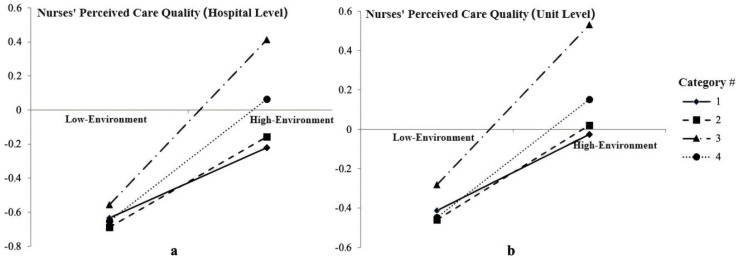
Slope graph of different profiles of occupational burnout: (**a**) nurses’ perceived care quality at the hospital level; (**b**) nurses’ perceived care quality at the unit level.

The results indicate that the nursing work environment has a positive impact on nurses’ perceived care quality (β = 0.206, *p* < 0.01; β = 0.193, *p* < 0.01), as shown in Figure 2. The interaction terms corresponding to Categories 2, 3, and 4 were all significant (*p* < 0.05), indicating the presence of moderation effects when Category 1 was used as the reference group (Appendix A). As illustrated in Figure 2, the moderating effects of different profiles of occupational burnout on the impact of the work environment on nurse-perceived care quality were ranked as follows: Category 3 > Category 4 > Category 2 > Category 1.

## 4. Discussion

### 4.1. Identifying Different Profiles of Nurse Burnout to Guide Targeted Interventions

This nationwide survey conducted in China identified four distinct profiles of occupational burnout among nurses, with the “Self-Fulfillment” and “Emotional Exhaustion” profiles being the most predominant, comprising 38.3% and 37.7% of respondents, respectively. Understanding the heterogeneity of nurses’ occupational burnout characteristics is crucial for healthcare institutions to devise targeted intervention strategies to address burnout [25]. This approach not only facilitates the identification of at-risk groups but also enables healthcare organizations to tailor their support systems and resources effectively [26]. By recognizing the specific demographic characteristics linked to each burnout profile, healthcare institutions can develop targeted interventions that consider factors such as age, work experience, education level, and work setting.

The “Self-Fulfillment” profile mainly consisted of clinical nurses aged 35 and above with over five years of service and educational levels at or below a bachelor’s degree. These nurses displayed minimal negative emotions at work and reported a sense of accomplishment in their nursing roles. This may be attributed to their rich clinical experience and higher stress-coping abilities, which enhance their self-efficacy and lead to a more positive perception of nursing quality [27]. Interestingly, this profile was notably concentrated in the western regions of China, which may be associated with a slower pace of life and lower stress levels in these areas [28].

On the other hand, more than half of the male nurses fell into the “Emotional Exhaustion” profile, which is consistent with previous studies by Chen et al. [29]. This suggests that gender discrimination and unfair treatment experienced by male nurses in clinical settings may contribute to burnout among this group [29,30]. Furthermore, nurses in this profile often held advanced academic qualifications and senior managerial roles, where decision fatigue and prolonged pressure associated with high responsibility could exacerbate burnout [31]. Such burnout can directly or indirectly affect the quality of nursing work and patient outcomes. Nurses with advanced educational qualifications are frequently expected to meet high expectations and take on extended responsibilities such as applying for research funding and preparing written documents [32]. Moreover, highly educated nurses may experience role ambiguity, high performance expectations from employers, and uncertainty about their professional contributions, particularly when not directly involved in clinical practice, all of which can contribute to psychological distress and occupational burnout [32,33].

The “Lack of Accomplishment” profile primarily included clinical nurses with five or fewer years of service who exhibited burnout specifically in the domain of personal accomplishment. This finding aligns with the research of Jiang Lili [34]. Inexperienced nurses, due to limited clinical exposure, are often tasked with simple and repetitive duties, which can hinder the cultivation of a professional identity and lead to stagnation. Furthermore, the absence of a structured feedback mechanism may dampen morale and reduce work enthusiasm [35]. The complexity of clinical work and inadequate guidance can also erode confidence levels and exacerbate burnout [36].

The “Self-Isolation” profile mainly consists of nurses working night shifts, most of whom are between 25 and 35 years old and come from the eastern coastal regions. These nurses typically display a detached attitude toward their work environment and career development opportunities. They lack active engagement in nursing tasks and maintain emotional distance from colleagues and patients [37]. This profile of nurse often has longer years of service but has not achieved career advancement. Prolonged work stress prompts them to develop defensive coping mechanisms, leading to emotional indifference and a sense of isolation at work [38]. A study indicates that nurses experience the most severe effort–reward imbalance, mental health impairment, and burnout risk among occupational groups facing mental stress, which exacerbates health issues and accelerates the burnout process [39].

### 4.2. Clarifying the Interacting Effects of Different Burnout Characteristics to Determine the Priority of Intervention Resources

Overall, the nursing work environment is a key factor influencing patient care quality, and this study’s findings are consistent with those of other research, gradually forming a consensus in the academic community [40,41]. A unique contribution of this study is the demonstration that different profiles of occupational burnout among nurses respond differently to the relationship between the nursing work environment and nurses’ perceived quality of care. Among the four profiles of nurses, those nurses classified as belonging to the “Self-Fulfillment” profile are the most sensitive to changes in the work environment, followed by those in the “Self-Isolation”, “Lack of Accomplishment”, and “Emotional Exhaustion” profiles. This variation may be related to differences in response mechanisms, resource acquisition, and utilization among nurses with distinct burnout profiles.

Improvements in the nursing work environment significantly influence the perceived quality of care among both Self-Fulfillment and Self-Isolation nurses. This suggests that optimizing working conditions may be the most effective approach to enhancing job satisfaction and care quality perception for these groups. Despite Self-Fulfillment nurses having the lowest levels of burnout and Self-Isolation nurses the highest, key factors in the work environment—such as compensation, career development opportunities, recognition, and leadership—have a substantial influence on the care quality for both groups [42,43]. For Self-Fulfillment nurses, management strategies should leverage their role as exemplars to foster a positive work culture and professional identity. By recognizing their achievements and creating a value-centered work environment, their motivation and career development can be further stimulated [44].

For nurses classified as the “Lack of Accomplishment” profile, who are primarily young and inexperienced, the moderating role in the relationship between the nursing work environment and perceived care quality is moderate. In China, clinical nurses with around five years of experience typically have opportunities to apply for positions such as education nurses, head nurses, or specialized nurses. However, due to limited vacancies and relatively narrow promotion channels, most nurses find it challenging to achieve advancement in professional titles or positions [45]. This lack of clear career development paths can lead to feelings of low accomplishment [46]. Furthermore, these nurses spend most of their time on the clinical frontlines and are in the early stages of their careers, where they are often still adapting to the demands of the profession [47]. Consequently, they may be more focused on mastering fundamental skills and meeting daily work challenges, which makes them less responsive to changes in the nursing work environment and less capable of utilizing available resources to enhance patient care quality. Therefore, for this group of nurses, in addition to actively fostering a healthy nursing work environment, it is crucial to provide targeted support. Implementing a mentorship program, where experienced nurses offer one-on-one guidance, can help young nurses better utilize the available resources in their environment, thereby allowing them to adapt more effectively to their roles and improve their work quality [48]. Additionally, establishing psychological support networks can provide a safe space for nurses to express their emotions, which, in turn, promotes emotional well-being and professional growth [49].

The “Emotional Exhaustion” profile is more common among senior nursing professionals and managers, who exhibit the lowest sensitivity to environmental changes. This could be related to their roles as leaders in implementing organizational changes. Externally, they are responsible for managing patient care and service quality; internally, they handle tasks like resource allocation and policy implementation. These responsibilities require them to quickly adapt and continuously anticipate and respond to organizational changes, which often keeps them in a high-stress environment, leading to sustained emotional and mental fatigue [50]. Typically, this group has strong self-regulation and coping skills, allowing them to maintain relative stability in the face of changes in the work environment. They are more likely to rely on their experience and abilities to manage environmental changes and external pressures. Moreover, the focus of managers and senior nursing professionals tends to be on strategic planning and team management rather than direct patient care, making them less sensitive to care quality issues than frontline nurses [51]. Therefore, for this group of nurses, it is crucial to focus on enhancing their management skills and ability to handle complex issues through leadership training. Optimizing work processes, e.g., by implementing intelligent management tools, can help reduce workload and repetitive tasks [52].

### 4.3. Implication for Policy and Practice

The findings of this study emphasize the need for healthcare institutions to adopt tailored intervention strategies based on distinct burnout profiles among nurses. Moreover, policymakers must recognize that different groups of nurses experience varying sensitivities to the relationship between the nursing work environment and patient care quality. Therefore, building on personalized interventions, it is important to prioritize intervention strategies to maximize management effectiveness. In particular, for the “Emotional Exhaustion” group, merely improving the work environment is insufficient; investigating the role of technological interventions, such as advanced monitoring systems and digital communication tools, in reducing workload and managing burnout could provide innovative and effective solutions for improving nurse well-being and patient care quality.

## 5. Conclusions

This study identifies four distinct profiles of nurses’ occupational burnout in the Chinese context and elucidates their varying moderating effects on the relationship between the nursing work environment and perceived patient care quality. These findings suggest the importance of tailoring strategies to address the specific needs of distinct nurse groups experiencing burnout. By employing personalized approaches, organizations can maximize management effectiveness, ultimately improving nurses’ psychological well-being, work performance, and patient care quality. However, this study has a few limitations. First, the data used were retrospective and derived from a cross-sectional study, which prevents the establishment of causal relationships between variables. Nevertheless, research has shown that there was little change in nurse burnout levels before and after the COVID-19 pandemic, and burnout was already quite severe before the pandemic, primarily due to structural pressures. Therefore, we believe that timeliness minimally affects the study’s results [53]. Furthermore, the large sample size enhances the robustness of the outcomes. Second, the sample was drawn exclusively from mainland China, limiting applicability across different cultural contexts. Future research should focus on longitudinal studies to explore causal relationships, on cross-cultural comparisons to understand cultural impacts, and on the effectiveness of specific interventions.

## Figures and Tables

**Table 1:** Fit indices for latent profile analysis of occupational burnout in Chinese Nurses (n = 16,382).

CAT	AIC	BIC	Entropy	LMRT	BLRT
1	356,932.725	356,978.949	-	-	-
2	350,345.763	350,422.802	0.709	<0.01	<0.01
3	346,347.933	346,455.788	0.727	<0.01	<0.01
4	344,076.533	344,215.204	0.781	<0.01	<0.01
5	342,181.079	342,350.565	0.764	<0.01	<0.01

**Table 2:** Comparison of different occupational burnout profiles across dimensions and total scores.

	Category 1 M (IQR)	Category 2 M (IQR)	Category 3 M (IQR)	Category 4 M (IQR)	H
Depersonalization	13 (11, 15)	5 (4, 8)	3 (1, 5)	21 (19, 24)	12,430.535 **
Personal Accomplishment	23 (18, 27)	31 (26, 35)	8 (4, 14)	18 (12, 25)	9761.05 **
Emotional Exhaustion	28 (23, 34)	17 (12.25, 22)	18.6 (0.16)	43 (37, 48)	6141.704 **
Burnout Total Score	63 (58, 69)	55 (49, 60)	31 (21, 41)	81 (73, 91.5)	11,672.093 **

Notes: Category 1 = Emotional Exhaustion Profile; Category 2 = Lack of Accomplishment Profile; Category 3 = Self-Fulfillment Profile; Category 4 = Self-Isolation Profile; ** *p* < 0.01.

**Table 3:** General demographic information across categories of nurse occupational burnout.

	Category 1 n (%)	Category 2 n (%)	Category 3 n (%)	Category 4 n (%)	χ^2^/*H*
Age					496.340 **
≤25	1891 (11.5)	670 (4.1)	1415 (8.6)	331 (2)	
25–35	3460 (21.1)	1595 (9.7)	3139 (19.2)	701 (4.3)	
35–45	694 (4.2)	387 (2.4)	1378 (8.4)	164 (1)	
>45	126 (0.8)	60 (0.4)	350 (2.1)	21 (0.1)	
Years of Service					698.751 **
≤5	3527 (21.5)	1358 (8.3)	2520 (15.4)	579 (3.5)	
5–10	1488 (9.1)	720 (4.4)	1494 (9.1)	346 (2.1)	
10–15	521 (3.2)	289 (1.8)	723 (4.4)	135 (0.8)	
15–20	337 (2.1)	162 (1)	618 (3.8)	85 (0.5)	
>20	298 (1.8)	183 (1.1)	927 (5.7)	72 (0.4)	
Gender					303.849 **
male	889 (5.4)	299 (1.8)	322 (2.0)	118 (0.7)	
female	5282 (32.2)	2413 (14.7)	5960 (36.4)	1099 (6.7)	
Marital Status					277.460 **
married	3284 (20)	1675 (10.2)	4242 (25.9)	687 (4.2)	
single	2769 (16.9)	1000 (6.1)	1954 (11.9)	511 (3.1)	
divorced	118 (0.7)	37 (0.2)	86 (0.5)	19 (0.1)	
Educational Level					281.087 **
technical secondary	256 (1.6)	150 (0.9)	273 (1.7)	48 (0.3)	
junior college	1767 (10.8)	850 (5.2)	1853 (11.3)	367 (2.2)	
bachelor	3445 (21)	1534 (9.4)	3897 (23.8)	720 (4.4)	
master's	574 (3.5)	145 (0.9)	241 (1.5)	66 (0.4)	
doctor	94 (0.6)	20 (0.1)	13 (0.1)	14 (0.1)	
postdoc	35 (0.2)	13 (0.1)	5 (<0.1)	2 (<0.1)	
Shift Method					449.178 **
night shift is required	4970 (30.3)	2112 (12.9)	4083 (24.9)	977 (6.0)	
night shift is not required	1201 (7.3)	600 (3.7)	2199 (13.4)	240 (1.5)	
Job Position					897.345 **
staff nurse	2905 (17.7)	1413 (8.6)	3724 (22.7)	841 (5.1)	
responsible team leader	823 (5)	451 (2.8)	786 (4.8)	128 (0.8)	
educational nurse	756 (4.6)	254 (1.6)	139 (0.8)	44 (0.3)	
head nurse	902 (5.5)	361 (2.2)	1087 (6.6)	117 (0.7)	
department head nurse	377 (2.3)	113 (0.7)	196 (1.2)	35 (0.2)	
nursing director	134 (0.8)	36 (0.2)	63 (0.4)	12 (0.1)	
nursing associate dean	66 (0.4)	11 (0.1)	4 (<0.1)	2 (<0.1)	
senior specialist nurse	123 (0.8)	30 (0.2)	129 (0.8)	18 (0.1)	
Title					409.689 **
junior	2339 (14.3)	1078 (6.6)	2999 (18.3)	665 (4.1)	
intermediate	2363 (14.4)	1160 (7.1)	2444 (14.9)	377 (2.3)	
associate senior	1047 (6.4)	355 (2.2)	565 (3.4)	103 (0.6)	
senior	247 (1.5)	75 (0.5)	99 (0.6)	27 (0.2)	
others	72 (0.4)	19 (0.1)	54 (0.3)	16 (0.1)	
unclassified	103 (0.6)	25 (0.2)	121 (0.7)	29 (0.2)	
Economic Region					72.905 **
old industrial base	430 (2.6)	140 (0.9)	284 (1.7)	66 (0.4)	
eastern coastal area	3594 (21.9)	1606 (9.8)	3597 (22)	680 (4.2)	
western	914 (5.6)	429 (2.6)	1189 (7.3)	208 (1.3)	
central	1233 (7.5)	537 (3.3)	1212 (7.4)	263 (1.6)	
Nursing Work Environment	108 (84, 129)	110 (89.25, 132)	112 (95, 129)	79 (61.5, 105)	810.778 **

Notes: Category 1 = Emotional Exhaustion profile; Category 2 = Lack of Accomplishment profile; Category 3 = Self-Fulfillment profile; Category 4 = Self-Isolation profile; ** *p* < 0.01.

## Data Availability

The data that support the findings of this study are available from the corresponding author upon reasonable request.

## Appendix A. Moderating Effect of Different Profiles of Occupational Burnout on the Relationship Between Work Environment and Perceived Care Quality

	**Nurses’ Perceived Care Quality** **(Hospital Level)**				**Nurses’ Perceived Care Quality** **(Unit Level)**			
	***β***	**se**	***t* Value**	**95% CI**	***β***	**se**	***t* Value**	**95% CI**
Constant	−0.428	0.086	−5.005 **	−0.596~−0.26	−0.220	0.087	−2.521 *	−0.391~−0.049
Nursing Work Environment	0.206	0.012	17.385 **	0.183~0.229	0.193	0.012	16.006 **	0.17~0.217
Lack of Accomplishment	0.005	0.021	0.224	−0.037~0.047	0.001	0.022	0.038	−0.042~0.043
Self-Fulfillment	0.356	0.017	20.528 **	0.322~0.39	0.344	0.018	19.473 **	0.31~0.379
Self-Isolation	0.133	0.035	3.820 **	0.065~0.202	0.072	0.036	2.035 *	0.003~0.142
Nursing Work Environment × Lack of Accomplishment	0.060	0.021	2.852 **	0.019~0.101	0.047	0.022	2.193 *	0.005~0.089
Nursing Work Environment × Self-Fulfillment	0.279	0.018	15.827 **	0.244~0.313	0.213	0.018	11.871 **	0.178~0.248
Nursing Work Environment × Self-Isolation	0.152	0.027	5.696 **	0.1~0.205	0.107	0.027	3.918 **	0.053~0.16
Age	−0.010	0.016	−0.598	−0.042~0.022	−0.021	0.017	−1.281	−0.054~0.011
Years of Service	0.000	0.010	0.023	−0.019~0.019	0.003	0.010	0.249	−0.017~0.022
Gender	0.114	0.025	4.621 **	0.066~0.162	0.076	0.025	3.031 **	0.027~0.125
Marital Status	0.002	0.016	0.141	−0.029~0.033	−0.012	0.016	−0.729	−0.043~0.02
Educational Level	0.082	0.010	7.939	0.062~0.102	0.045	0.011	4.290 **	0.025~0.066
Shift Method	−0.074	0.018	−4.001 **	−0.11~−0.038	−0.077	0.019	−4.131 **	−0.114~−0.041
Job Position	−0.026	0.004	−6.005 **	−0.034~−0.017	−0.012	0.004	−2.807 **	−0.021~−0.004
Title	−0.001	0.006	−0.133	−0.012~0.01	0.012	0.006	2.050 *	0.001~0.023
Economic Region	−0.043	0.008	−5.203 **	−0.059~−0.027	−0.049	0.008	−5.843 **	−0.066~−0.033
F value	201.5045				154.7877			
R	0.4057				0.3626			
R^2^	0.1646				0.1314			

Notes: * *p* < 0.05, ** *p* < 0.01.
